# Correction: The oldest unvaccinated Covid-19 survivors in South America

**DOI:** 10.1186/s12979-022-00319-3

**Published:** 2022-12-07

**Authors:** Mateus V. de Castro, Monize V. R. Silva, Michel S. Naslavsky, Marilia O. Scliar, Kelly Nunes, Maria Rita Passos-Bueno, Erick C. Castelli, Jhosiene Y. Magawa, Flávia L. Adami, Ana I. S. Moretti, Vivian L. de Oliveira, Silvia B. Boscardin, Edecio Cunha-Neto, Jorge Kalil, Emmanuelle Jouanguy, Paul Bastard, Jean-Laurent Casanova, Mauricio Quiñones-Vega, Patricia Sosa-Acosta, Jéssica de S. Guedes, Natália P. de Almeida, Fábio C. S. Nogueira, Gilberto B. Domont, Keity S. Santos, Mayana Zatz

**Affiliations:** 1 Human Genome and Stem Cell Research Center, University of São Paulo, São Paulo, São Paulo Brazil; 2 Department of Genetics and Evolutionary Biology, Biosciences Institute, University of São Paulo, São Paulo, São Paulo Brazil; 3 Department of Pathology, School of Medicine, UNESP - São Paulo State University, Botucatu, São Paulo Brazil; 4 Laboratório de Imunologia, Instituto do Coração (InCor), LIM19, Hospital das Clínicas da Faculdade de Medicina da Universidade de São Paulo, (HCFMUSP), São Paulo, Brazil; 5 Instituto de Investigação em Imunologia-Instituto Nacional de Ciências e Tecnologia-iii-INCT, São Paulo, Brazil; 6 Departamento de Clínica Médica, Disciplina de Imunologia Clínica e Alergia, Faculdade de Medicina da Universidade de São Paulo, São Paulo, Brazil; 7 Laboratory of Antigen Targeting to Dendritic Cells, Department of Parasitology, Institute of Biomedical Sciences, University of São Paulo, São Paulo, Brazil; 8 Laboratory of Human Genetics of Infectious Diseases, Necker Branch, INSERM U1163, Necker Hospital for Sick Children, Paris, France; 9 Imagine Institute, University of Paris, Paris, France; 10 St. Giles Laboratory of Human Genetics of Infectious Diseases, Rockefeller Branch, The Rockefeller University, New York, NY USA; 11 Proteomics Unit, Department of Biochemistry, Institute of Chemistry, Federal University of Rio de Janeiro, Rio de Janeiro, Brazil; 12 Laboratory of Proteomics (LabProt), Institute of Chemistry, LADETEC, Federal University of Rio de Janeiro, Rio de Janeiro, Brazil

**Correction: Immun Ageing 19, 57 (2022)**

**https://doi.org/10.1186/s12979-022-00310-y**

Following publication of the original article [1], the authors identified an error in the author name of Jéssica de S. Guedes.

The incorrect author name is: Jéssica S. de Guedes

The correct author name is: Jéssica de S. Guedes

The author group has been updated above and the original article [1] has been corrected.
